# Controlling magnetic transition of monovacancy graphene by shear distortion

**DOI:** 10.1038/s41598-017-01881-3

**Published:** 2017-05-11

**Authors:** Fei Gao, Shiwu Gao

**Affiliations:** Beijing Computational Science Research Center, ZPark II, 100193 Beijing, China

## Abstract

The effect of shear distortion on the vacancy induced magnetism in graphene is investigated using extensive first-principles calculations. It is found that shear distortion can lead to magnetic transition between two states with high and low magnetic moments. Such a transition is reversible and results from the breaking of the in-plane symmetry of the local atoms, which reverses spin polarization of the π bands of the vacancy states near the Fermi level and leads to the change of magnetic transition by 1 µ_B_. This finding opens the possibility for nanomechanical control of graphene magnetism and has potential applications in spintronics and magnetic sensing.

## Introduction

Introducing magnetism into graphene, which is intrinsically a nonmagnetic 2D material, has been the endeavor of many ongoing researches^1–9^. Magnetism of graphene can be created in several different ways by for example atom vacancies^10–12^, adsorption of magnetic atoms^13, 14^ and recently by hydrogen modifications^15, 16^. Among them, single atom vacancies break the local bonding symmetry of the *sp* ^2^ states in the pristine lattice and introduce dangling bonds at the vacancy site. It generates spin polarization in the valence bands near the Fermi level, resulting in local magnetism of the graphene lattice. It has been known that the ground state of the monovacancy graphene is a ferromagnetic state with a magnetic moment of 1~1.7 µ_B_ depending on the vacancy concentration^17–23^. Local spin polarization of the graphite^24^ and graphene^25^ vacancy can be probed by spin-polarized scanning tunneling microscope.

In light of any potential application in spintronic devices and quantum information, it would be highly desirable to achieve dynamical and reversible control of the local magnetization of the graphene. In essence, this depends on how the local spin polarization of the graphene lattice couple and respond to external perturbations^26^ applied. It has recently been proposed that the spin channels of graphene nanoribbons can be modified using single tetragon molecules^27^. Nano-mechanical and vibrational coupling^26^ would also be interesting to explore. So far, the control and manipulation of graphene magnetism has not yet been well understood.

**Figure 1:**
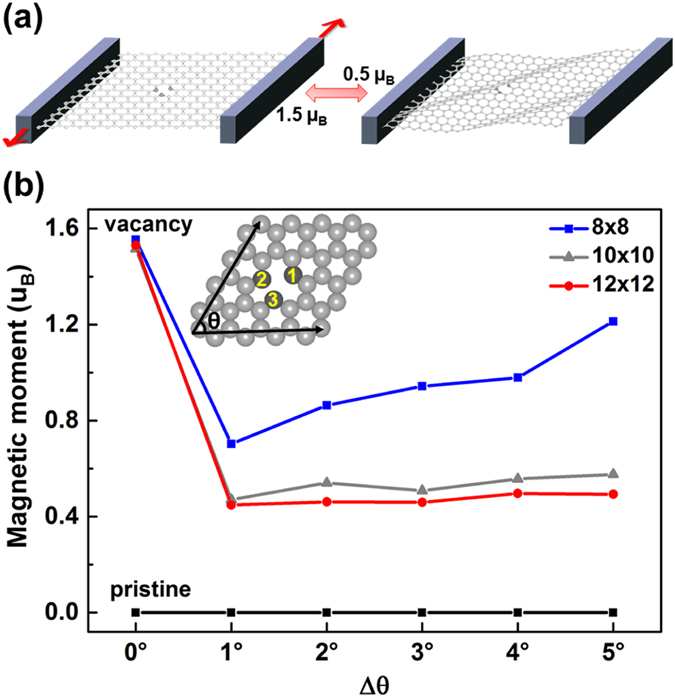
Variation of magnetism of the monovacancy graphene by shear distortion. (**a**) Schematic diagram demonstrating reversible magnetic transition controllable by shear distortion in monovacancy graphene; (**b**) The magnetic moment as a function of deformation angle Δ*θ*. Blue (8 × 8 graphene), gray (10 × 10 graphene) and red (12 × 12 graphene) lines represent the magnetic moment with single vacancy in graphene, and the black line represents the magnetic moment in pristine graphene. The insert shows the geometry of the calculated supercell with initial angle *θ* = 60°. The magnetic moment undergoes a sharp and reversible transition at Δ*θ* = 1°.

Here we propose and demonstrate a robust way to control the graphene magnetism using nanomechanical deformation, based on extensive and rigorous density functional theory (DFT) calculations. It is found that shear deformation of graphene with single-atom vacancy can lead to an electronic transition between two magnetic states with high and low magnetic moments (Fig. 1(a)). Such a transition is reversible and occurs when the shear angle is deformed by less than 1 degree and remains stable in a wide range of torsional angles. Detailed analysis of the local distortion and spin density distribution reveals that this magnetic transition is associated with the shift of the σ band and the reversal of the spin polarization of the π bands of the vacancy states. Dynamical control of the magnetism has general implications to the fundamental understandings and potential applications in spintronics and magnetic sensing using graphene based materials.

## Results

Our supercell calculations performed here are aimed to simulate isolated vacancies with ferromagnetic solutions, although couplings between vacancies can compete, according to the Lieb’s theorem^28^, and lead to antiferromagnetic states. In the small concentration limit, however, it is well known that the ground state of monovacancy graphene is a ferromagnetic state, and its relaxed geometry is nearly flat as shown in the insert of Fig. 1(b). In the plane, the threefold symmetry (C_3_) of the pristine lattice is broken due to Jahn-Teller distortion^20, 29, 30^ and re-bonding between two of the nearest neighbor carbon atoms (atoms 2 and 3 as labeled in Fig. 1(b)). The calculated magnetic moment of this state is 1.53 µ_B_ in the 12 × 12 supercell. The optimized geometry and the calculated magnetic moment agree well with previous calculations in the literature^18–23^.

### Magnetic moment

Starting from the ground state and its spin configuration, we next investigate how shear distortion modifies the geometry and magnetism of the monovacancy graphene. Such a shear distortion could possibly be implemented experimentally by piezoelectric device in suspended graphene as sketched schematically in Fig. 1(a), or through other nanomechanical coupling methods^26^. The shear deformation is introduced by a torsional deformation angle, Δ*θ*, into the supercells and then structure optimization is carried out to find the new lowest energy state under the deformation. This step is time consuming at large deformation angles, when the final structure differs substantially from that of the ground state. To ensure that the final state is the lowest energy state and unique for the given Δ*θ*, many repeated calculations have been done starting from different initial geometries and spin configurations. Different initial Jahn-Teller distortion orientation does not change the results, and they end up in the same final direction, which is shown in Fig. 1(b), as the torsional deformation angle increases. Figure 1(b) displays the calculated magnetic moment as a function of Δ*θ*. At a small deformation, such as Δ*θ* = 1°, the magnetic moment jumps from 1.53 to 0.45 µ_B_ in the 12 × 12 supercell and keeps almost constant at large deformation angles. In smaller supercells such as 8 × 8, the jump is smaller, from 1.55 to 0.70 µ_B_ and the magnetic moment increases slightly from 0.7 to 1.21 µ_B_ as Δ*θ* varies from 1 to 5°. This is due to fact that the interaction between vacancies exists and the stress of the deformation cannot be fully relaxed in small supercells. We have also done calculations using the 10 × 10 supercell and obtained similar results as those of the 12 × 12 case. Therefore, the deformed states of the 12 × 12 supercell, on which we base all of following analyses and discussions, is fully relaxed and converged. We can also point out that low-quality DFT calculations can yield inaccurate magnetic moment for the low magnetic state with vanishing magnetic moment. However, our extensive calculations can rule out such a nonmagnetic state at the given range of vacancy concentration. In contrast, when the same deformation is applied to pristine graphene, it remains nonmagnetic under all degrees of lattice deformation as shown by the black solid line in Fig. 1(b). This implies that shear distortion, though inducing magnetic transition, is neither the origin of the vacancy magnetism nor its transformation.

### Spin density distribution

**Figure 2:**
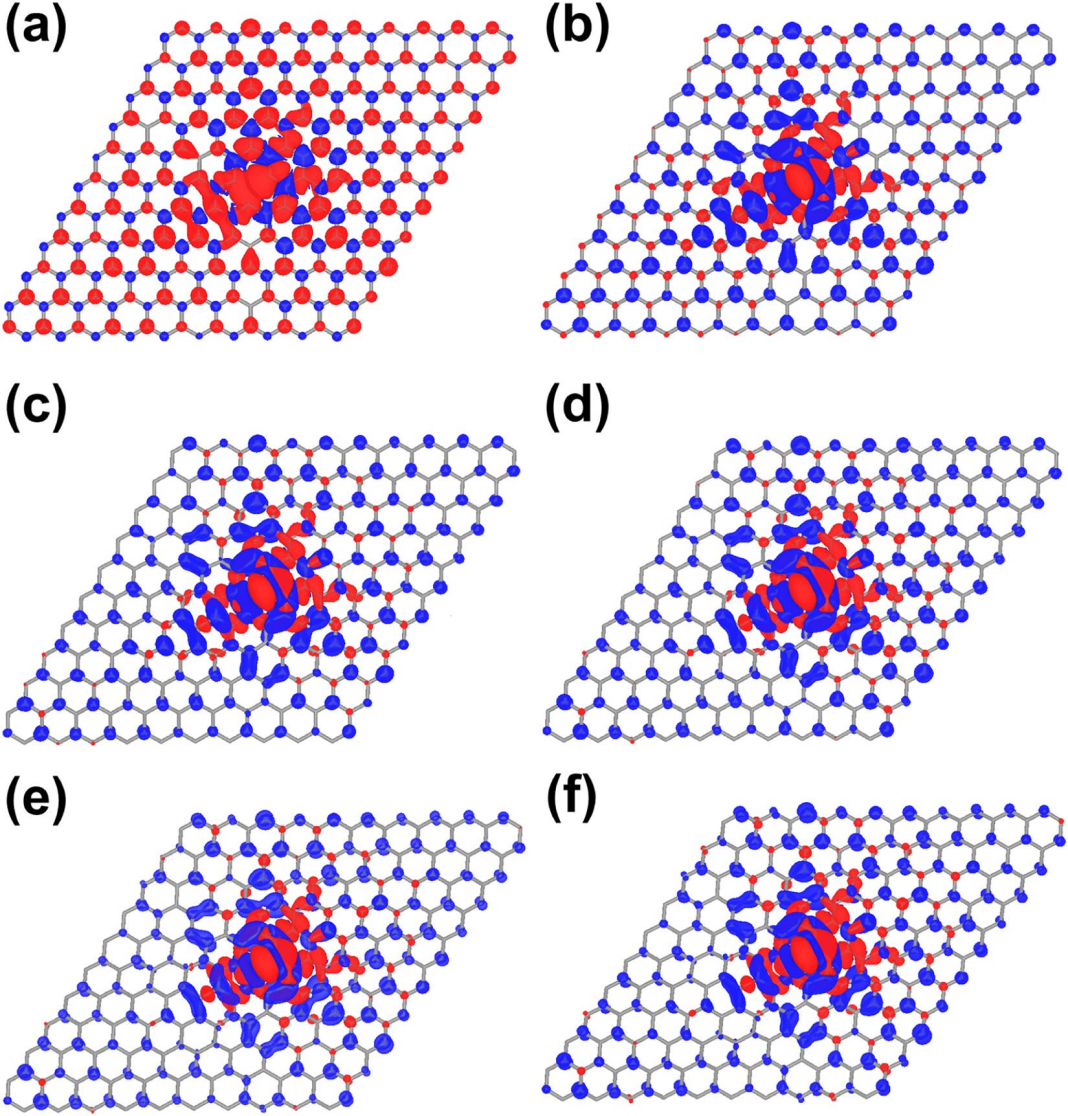
Evolution of spin density distribution of graphene with monovacancy in the 12 × 12 supercell. (**a**)–(**f**) at Δ*θ* = 0°–5°. Isosurfaces with values of ±0.0001 e/bohr^3^ are shown. The red and blue surfaces correspond to the densities of spin up and spin down states, respectively.

It is interesting to find out how this magnetic jump happens in space across the lattice. Figure 2 shows the evolution of spin density distribution as a function of Δ*θ* ranging from 0 to 5 degrees. The red and blue isosurfaces correspond to the spin up and spin down densities at 0.0001 e/bohr^3^, respectively. In the unperturbed graphene, Fig. 2(a) (Δ*θ* = 0°), the spin up density (the red surface) is dominantly distributed at the vacancy site and decays slowly away from the vacancy. Consistent with the magnetic moment, there is a change of color map at Δ*θ* = 1° on most atomic sites, as the lattice is slightly deformed. It then remains almost the same at larger angles (1–5° as shown in panels (b)–(f))). Figure 2 clearly shows that there is a spin transition on most lattice sites at 1°. We like to point out that such a spin transition occurs in both spin up and spin down configurations and is independent of the initial orientation of magnetization. The deformation leads to partial cancellation between spin-up and spin-down densities around the vacancy site, and an overall weaker spin polarization in the whole supercell. Moreover, the spin density distribution is rather extended, suggesting that magnetic transition is long-ranged, which is in line with recent experimental observations.

### Deformed geometry

**Figure 3:**
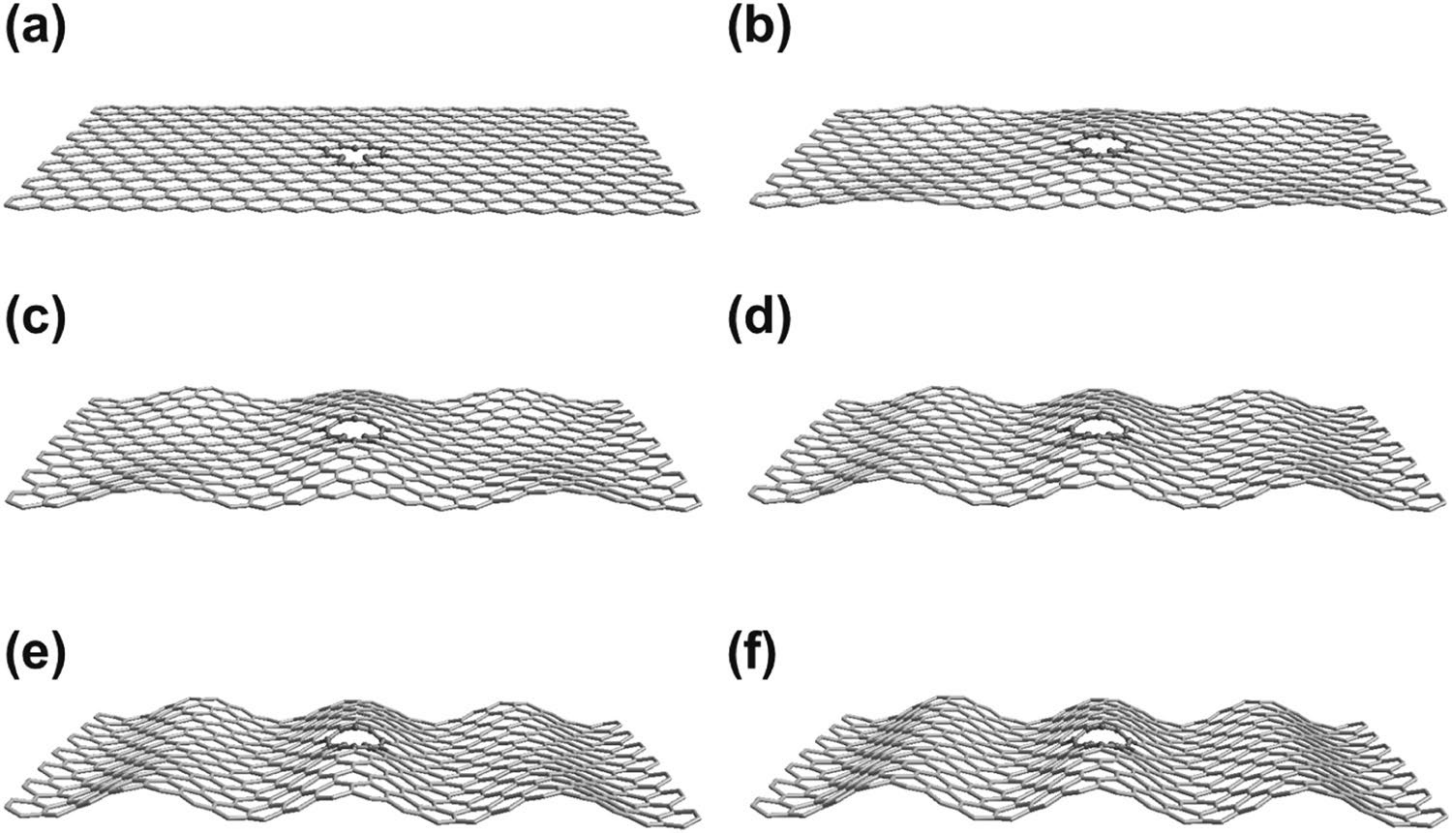
The optimized geometries of a single vacancy in graphene under shear distortion. (**a**)–(**f**) For Δ*θ* = 0°–5°, respectively.

Figure 3 displays the evolution of atomic structure as a function of Δ*θ*. These structures are obtained by fully relaxing all atomic positions under the given distortion. Panel (a) is the structure of the monovacancy graphene in the 12 × 12 supercell without distortion. The lattice shows overall flat. Within the plane, atoms 2 and 3 contract slightly and rebond via the Jahn-Teller distortion with a distance between atoms 2 and 3 of 1.93 Å^20, 29, 30^. The lattice geometries at Δ*θ* = 1° and 2° have the same shape and the lattice forms a wrinkled structure (Fig. 3(b–c)) with atom 1 protruding a height of *h* = 1.78 Å, relative to the graphene sheet at Δ*θ* = 1° and *h* = 2.11 Å at Δ*θ* = 2°, respectively. As Δ*θ* increases, the graphene lattice starts to form corrugated stripes from Δ*θ* = 3° as shown in Fig. 3(d), and keeps the same shape at larger angles. The vacancy is found on the wave top of the strips with *h* = 2.40, 2.77 and 2.88 Å for Δ*θ* = 3°, 4° and 5°, respectively. Moreover, atoms 2 and 3 are moving closer, and the vertical height between atom 1 and atom 2/3 increases from 0.64 to 0.82 Å when Δ*θ* varies from 1 to 5°, as shown in more detail in the Supplementary Figure S6. The increasing distortion of the stripes at larger angles, however, do not alter the induced magnetic moment, 0.45 µ_B_, as shown in Fig. 1.

Interestingly, the structural transformation, from wrinkles to stripes at Δ*θ* = 3°, does not coincide with the magnetic transition that occurs at Δ*θ* = 1°. The pristine graphene also undergoes the same wrinkle-stripe transition under the shear distortion (shown in the Supplementary Fig. S1). However, it remains nonmagnetic under all Δ*θ* as shown by the black line in Fig. 1(a). This means that the magnetic transition in Fig. 1 is not directly correlated with the geometry transformation. Instead, the magnetic transition is originated from the local symmetry breaking (R2 reflection symmetry) of the vacancy geometry, which is triggered by the shear deformation.

### Electronic structure

**Figure 4:**
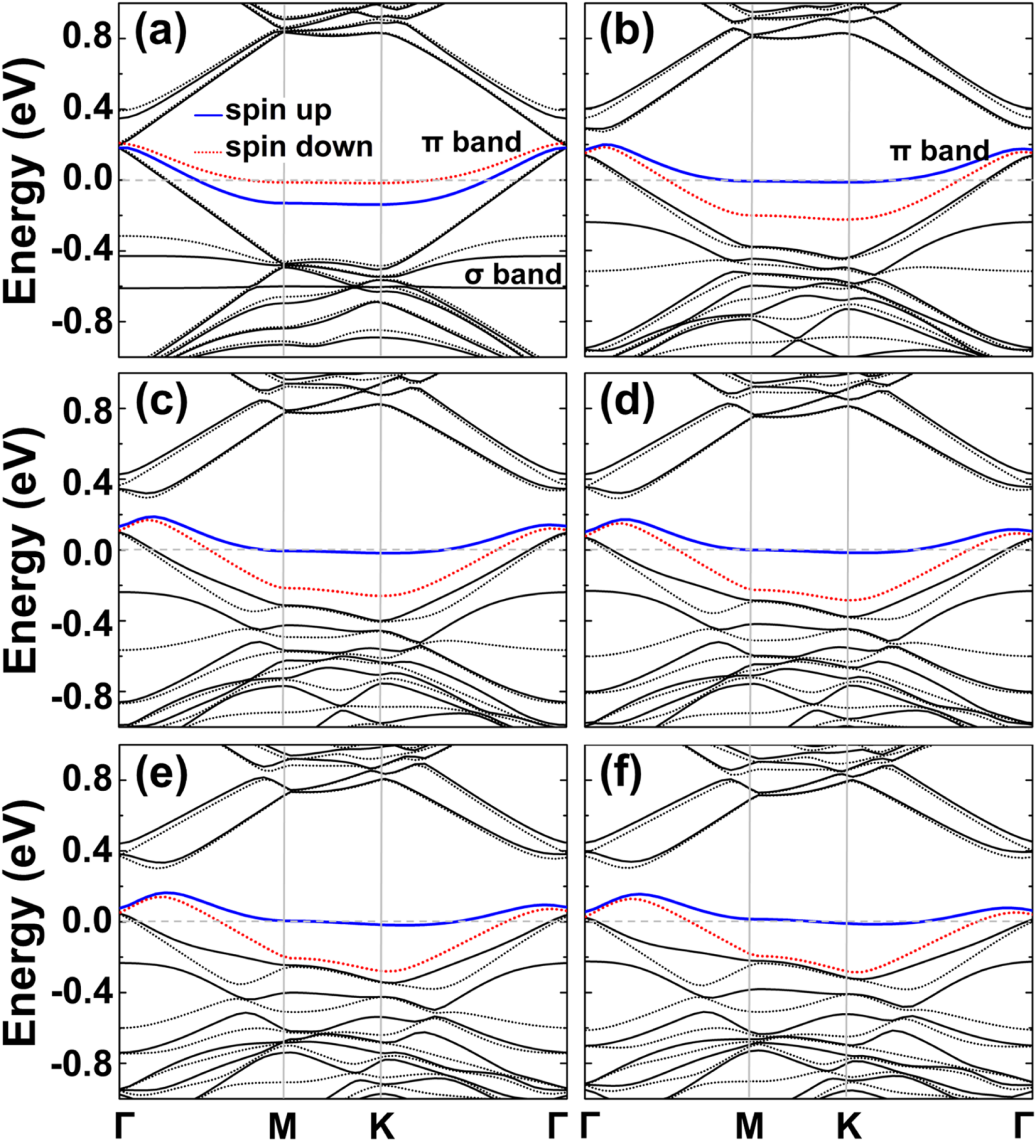
Band structures of the monovacancy in the 12 × 12 supercell. (**a**)–(**f**) At Δ*θ* = 0°–5°, respectively. Here, (**a**) Δ*θ* = 0° and (**b**) Δ*θ* = 1° correspond to the two magnetic states near the transition point.

To find out the origin of the magnetic transition, we analyzed in detail the electronic structure of the defect graphene upon distortion. Figure 4 displays the evolution of the band structure as a function of Δ*θ*. At the transition point Δ*θ* = 1°, the π bands near the Fermi level (blue solid line and red dotted line) shift in opposite directions. More specifically, the spin up state of the π bands shifts upwards from −0.131 to −0.008 eV at the M point and from −0.135 to −0.012 eV at K, and from 0.182 to 0.170 eV at Γ. In contrast, the spin down band shifts oppositely from 0.210 to 0.152 eV at Γ and from −0.017 (−0.014) to −0.225 (−0.219) eV at M (K) when *θ* goes from 0° to 1°. Simultaneously, one σ band located at −0.61 eV of the vacancy states, which is associated with the in-plane dangling bond of atom 1, disappears from the valence band gap at Δ*θ* = 1° and is shifted downward to the valence band. Such a downward shift indicates a large interaction yet does not contribute to the change of magnetism. Instead the change of magnetic moment results mostly from the reversal of spin polarization of the π bands of vacancy states near the Fermi level. For the planar structure at undeformed state, the total magnetic moment of 1.53 µ_B_ is the sum of the σ band of 1 µ_B_ and the extended π orbitals of 0.53 µ_B_. In contrast, the transformed σ band still provides the magnetic moment of 1 µ_B_, reversal of π band spin polarization gives a negative value of −0.5 µ_B_, leading to a low magnetic state ~0.45 µ_B_. The reversal of spin polarization of the π band is thus mainly responsible for the jump of the total magnetic moment by 1 µ_B_. From the analysis of local density of states (LDOS), it can be confirmed that this σ band is dominantly localized on atom 1 at Δ*θ* = 0° as it shows no energy dispersion. It becomes delocalized under distortion, but does not contribute to the change of the total magnetic moment because it is occupied before and after the transition. Instead, the opposite shift of the π bands is responsible for the long-range spin transition across the lattice, as shown by the long-range spin density distribution shown in Fig. 2. When the magnetic transition occurs, the band structures do not change appreciably as Δ*θ* further increases. In particular, there is no change of spin polarization when the geometry transforms at Δ*θ* = 3°. It suggests local bonding symmetry of the atoms around the vacancy is mainly responsible for the magnetic transition. This also suggests that magnetic transition could also be induced by other external deformations.

## Discussion

We have investigated the effect of shear distortion on the vacancy induced magnetism of graphene, based on extensive first-principles calculations, and found a reversible transition between two distinct magnetic states with high and low magnetic moments. This transition results from a mechanical response to shear distortion and is associated with the local symmetry breaking of the vacancy. It opens the possibility of dynamical control of graphene magnetism via manipulation of lattice deformation. We believe that such a reversible control is versatile and could find potential applications in spintronics and magnetic sensing. In light of practical application, it would be desirable to investigate possible long-range ordering, in particular the competition and stability of possible antiferromagnetic ordering as implied by the Lieb’s theorem^28^. These issues will be pursued in future investigations. This finding has thus general implications to the nanoscale control of graphene magnetism using defects and other related materials.

## Methods

Our DFT calculations are performed using Vienna *Ab initio* Simulation Package (VASP)^31^. The projector augmented-wave (PAW)^32, 33^ pseudopotentials and the generalized gradient approximation (GGA)^34^ in Perdew-Burke-Ernzerholf form^35^ for the exchange-correlation energy are employed. Spin polarization is invoked in all calculations, and spin-orbital coupling can be neglected in graphene. Most calculations are carried out with an energy cutoff of 600 eV in the plane wave basis set, and the main results are also double checked with 800 eV cutoff. The supercells contain a vacuum layer ~20 Å and all atoms are relaxed until the forces on each atom are less than 0.01 eV/Å. The C-C bond length of pristine graphene is found to be 1.424 Å, which is consistent with the experimental value of 1.42 Å. The structural and electronic properties of the monovacancy defect are calculated using the 8 × 8 and 10 × 10, and 12 × 12 supercells. The Brillouin zone is integrated using the Monkhorst-Pack scheme with a 3 × 3 × 1 mesh sampling, and checked with larger (6 × 6 × 1) k-mesh to ensure convergence.

## Electronic supplementary material

Controlling magnetic transition of monovacancy graphene by shear distortion
