# Supplementary material for: Controlling magnetic transition of monovacancy graphene by shear distortion
Source: Sci Rep. 2017 May 11;7:1792. doi: 10.1038/s41598-017-01881-3 (PMC5431955; doi:10.1038/s41598-017-01881-3)
Supplement: Supplementary file 1 — Controlling magnetic transition of monovacancy graphene by shear distortion [file 41598_2017_1881_MOESM1_ESM.pdf]

Supplementary information: Controlling magnetic transition of monovacancy graphene by shear distortion

*Fei Gao and Shiwu Gao**
Beijing Computational Science Research Center, ZPark-II, 100193, Beijing, China
*swgao@csrc.ac.cn

Supplementary Figures

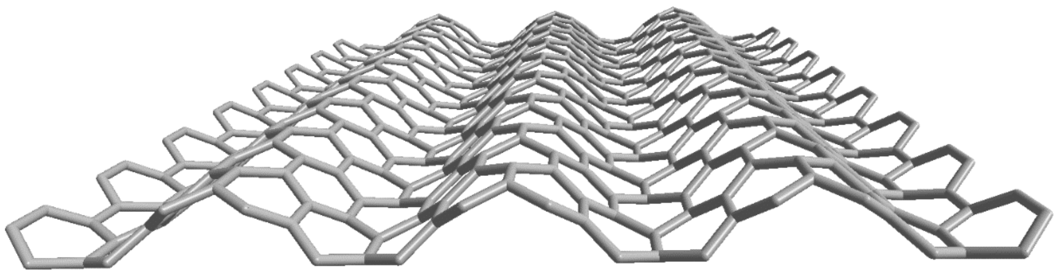

Supplementary Figure S1. The optimized structures of the pristine graphene after shear distortion.

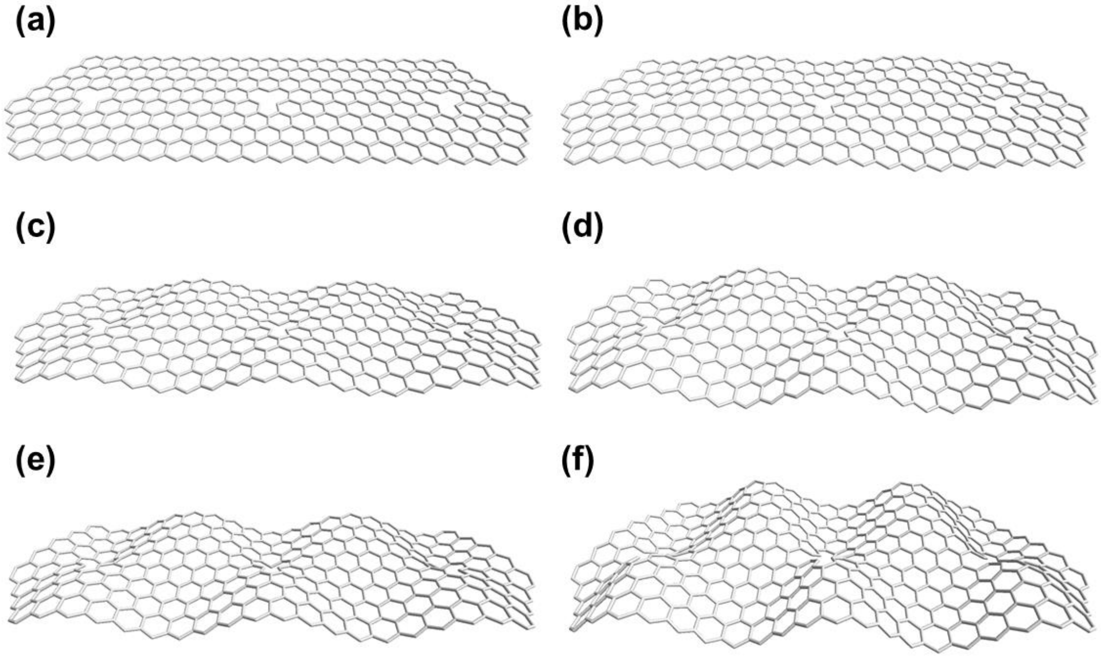

Supplementary Figure S2. The optimized geometry of the monovacancy in the 8×8 graphene at (a) – (f) $\Delta\theta$ = 0º - 5º. These structures are different from those obtained with larger supercells, which explains why magnetic moment also differs.

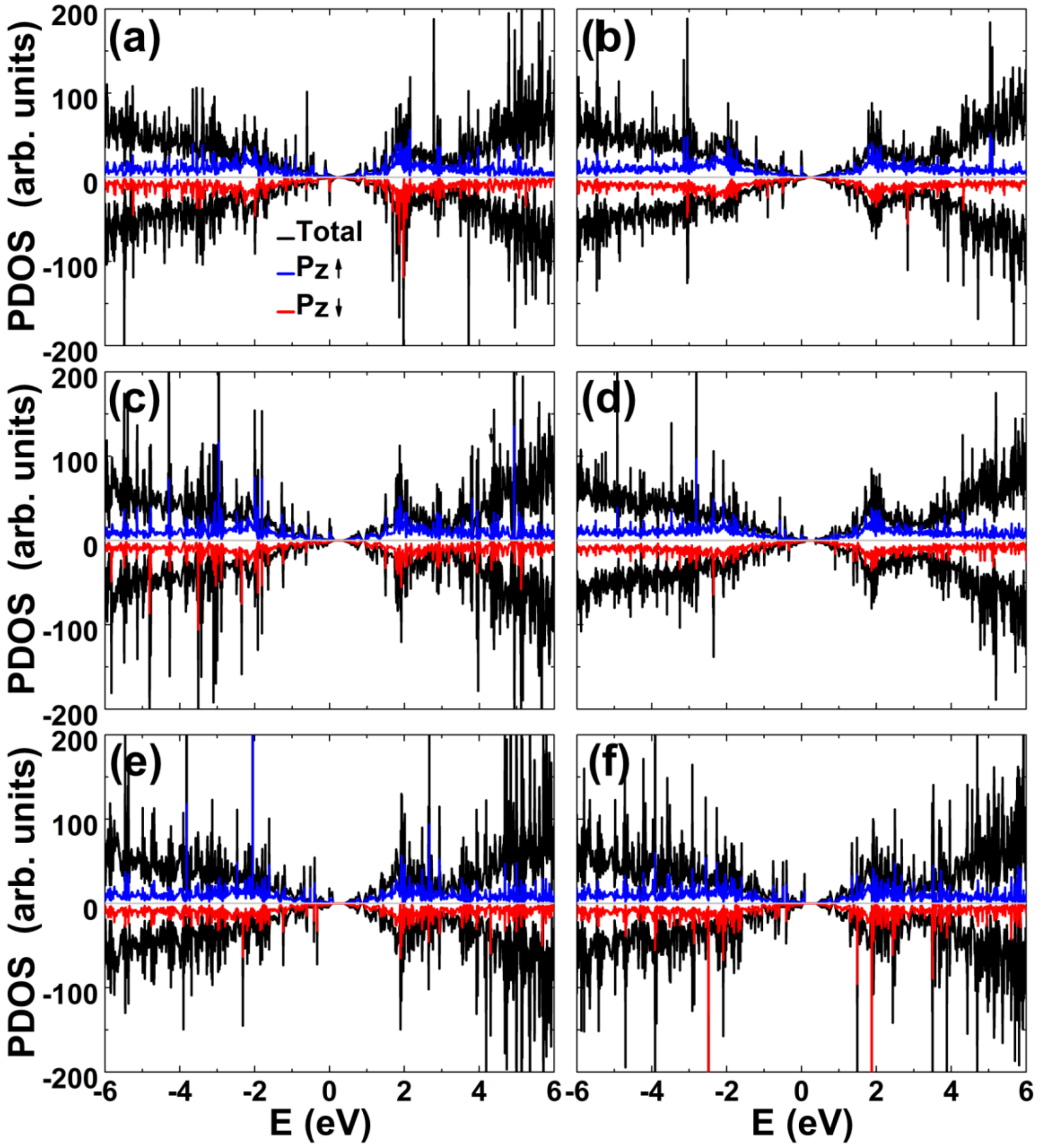

Supplementary Figure S3. Partial density of states (PDOS) of the monovacancy graphene in the 12×12 supercell at $\Delta\theta$ = 0º - 5 º, (a) – (f).

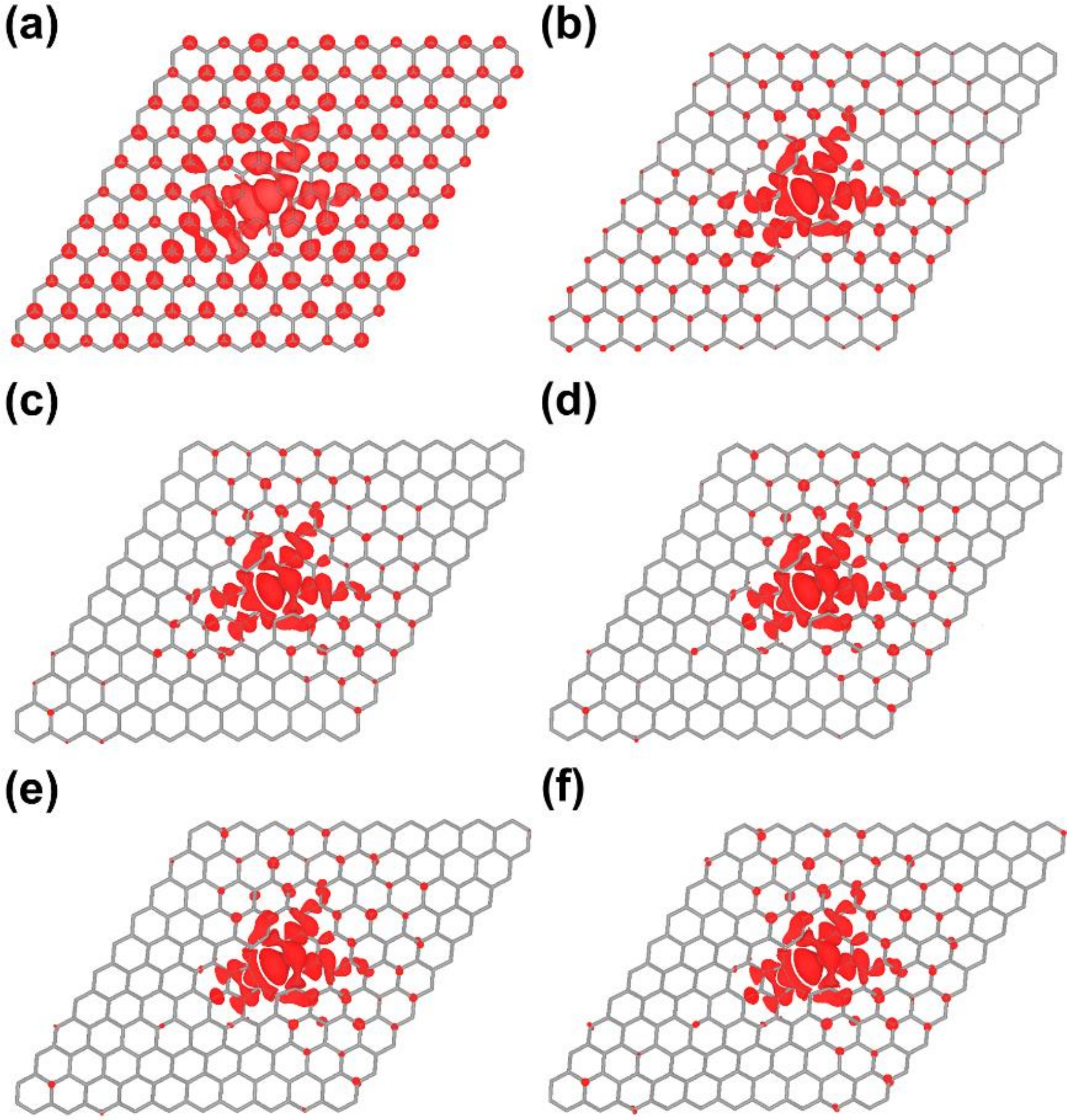

Supplementary Figure S4. Spin density of the graphene with single-atom vacancy (12×12 supercell) at (a) – (f) $\Delta\theta$ = 0º - 5º. Isosurfaces with values of +0.0001 eV/bohr^3 are shown. The red surfaces correspond to the densities of spin up.

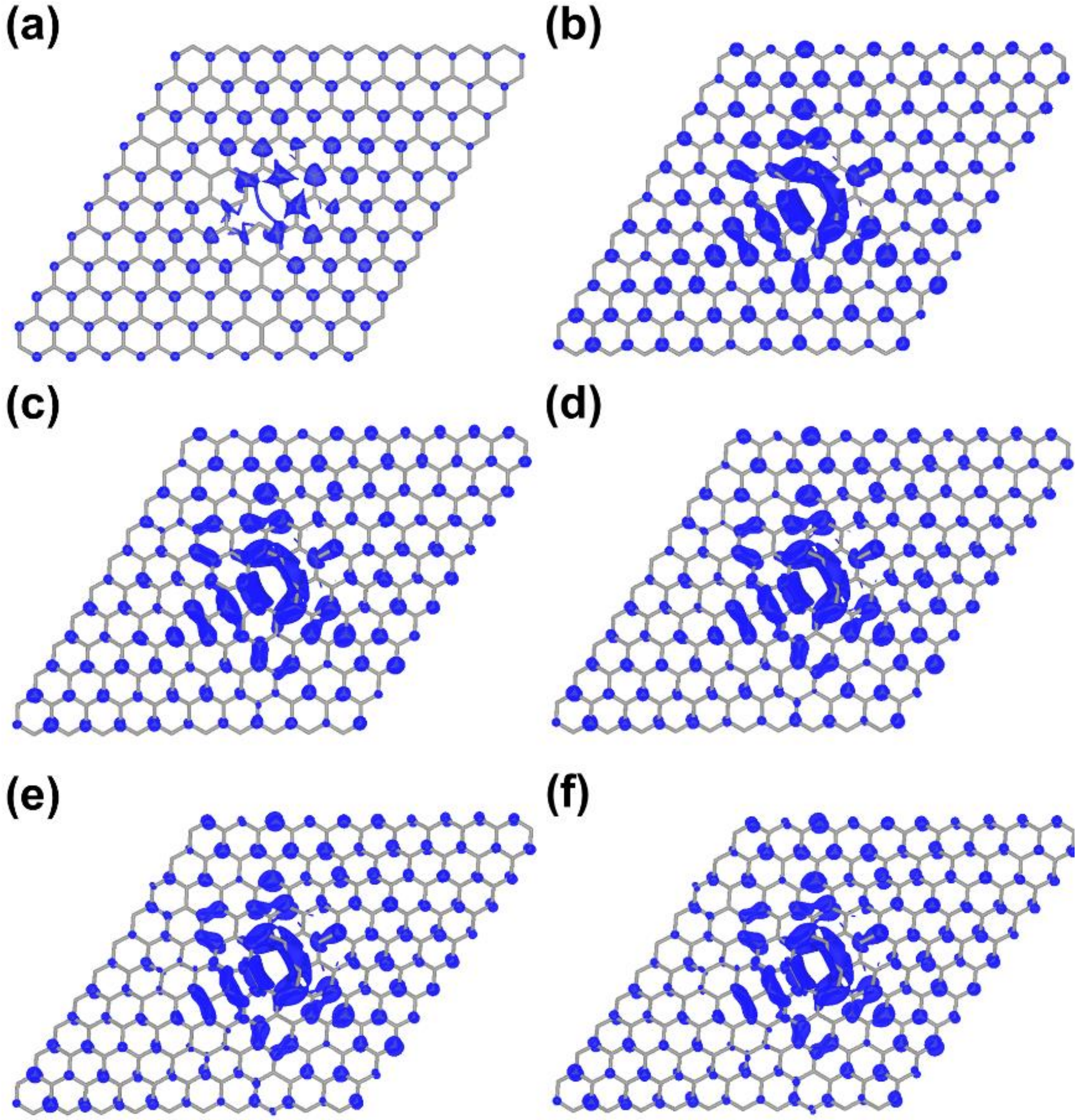

Supplementary Figure S5. Spin density of the graphene with single-atom vacancy (12×12 supercell) at (a) – (f) $\Delta\theta$ = 0º - 5º. Isosurfaces with values of -0.0001 eV/bohr^3 are shown. The blue surfaces correspond to the densities of spin down states.

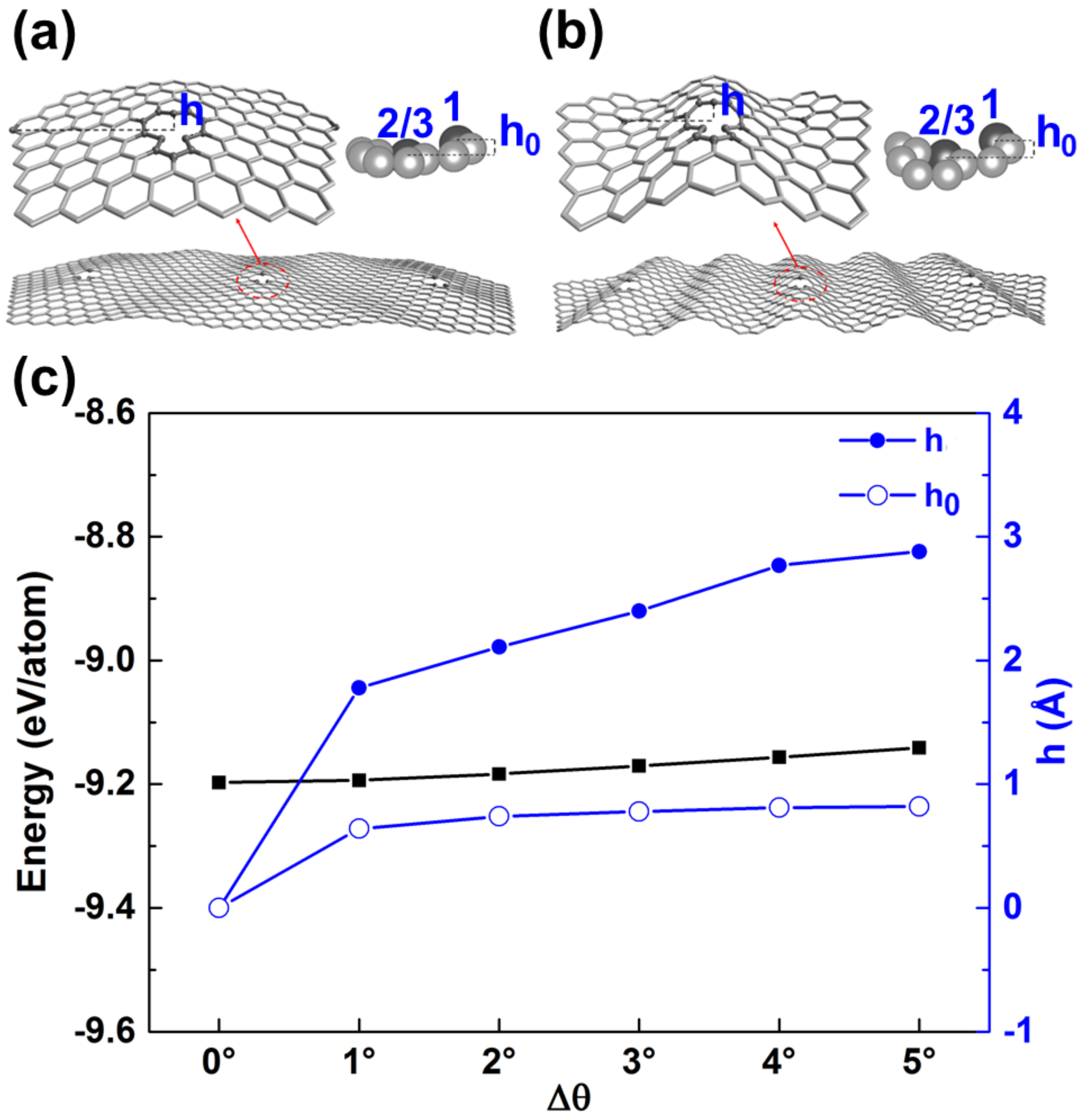

Supplementary Figure S6. The optimized geometry of the monovacancy graphene in the 12×12 supercell at (a) $\Delta\theta$ = 1º-2º; (b) $\Delta\theta$ = 3º-5º; (c) The angular dependence of the energy per atom and vertical heights of deformation for the 12×12 supercell. Here, h denotes the thickness of the deformed graphene sheet, and h_0 represents the vertical distance between atoms 1 and 2(3).
